# International medical graduates (IMGs) matching into US orthopaedic surgery residency: a fifteen year analysis of trends in applications and geographical distribution

**DOI:** 10.1007/s00264-024-06283-5

**Published:** 2024-09-02

**Authors:** Amir Human Hoveidaei, Natalie M. Kistler, Garrett Jackson, Dawn M. LaPorte, Jorge A. Chahla, Nathanael D. Heckmann

**Affiliations:** 1 International Center for Limb Lengthening, Rubin Institute for Advanced Orthopedics, Sinai Hospital of Baltimore, Baltimore, MD USA; 2 Department of Orthopaedic Surgery, Keck School of Medicine, University of Southern California, Los Angeles, CA USA; 3 Department of Orthopaedic Surgery, Rush University Medical Center, Chicago, IL USA; 4 Department of Orthopaedic Surgery, The Johns Hopkins University, Baltimore, MD USA; 5 Department of Orthopaedic Surgery, Keck Medical Center of USC, 1520 San Pablo St, Ste 2000, Los Angeles, CA 90333 USA

**Keywords:** Foreign medical graduates, International medical graduates, National residency match program, Research, Residency applications, Selection criteria

## Abstract

**Purpose:**

International Medical Graduates (IMGs) face challenges in securing orthopaedic surgery residencies in the U.S. This study examines residency matching trends and geographic distribution for U.S. and non-U.S. citizen IMGs.

**Methods:**

The National Resident Matching Program (NRMP) data from 2008 to 2022 were analyzed for USMLE scores, publication counts, and match rates, using linear regression. The proportion of non-US IMGs in orthopaedic surgery residency was compared with the foreign-born population of each region based on the 2021 American Community Survey.

**Results:**

The overall IMG fill rate decreased significantly from 2.04% in 2008 to 1.26% in 2022 (*P* = 0.002). The number of publications for matched US IMGs was at least three times that of matched MD seniors and about two times that of unmatched US IMGs. Matched non-US IMGs had approximately five and three times the number of publications as matched MD seniors and unmatched non-US IMGs, respectively. Mississippi had the highest IMG-to-all-filled-position ratio (6.7%) and New York matched the most IMGs (36 residents). Although the foreign-born population comprises approximately 13.72% of the US population, non-US IMGs accounted for less than 1% of total matched residents. When compared to the foreign-born population, non-US IMGs were underrepresented in the US. This underrepresentation was observed in all nine geographic divisions, particularly in the West South Central and Pacific regions.

**Conclusions:**

While IMGs constitute a low percentage of matched MDs in orthopaedic surgery, they show three to five times more publications than MD seniors. IMGs should recognize the importance of higher publication numbers in the matching process as well as states with higher IMG matching rates.

## Introduction

International medical graduates (IMGs) are physicians trained outside the country where they seek licensure. They are categorized as US IMGs if they are U.S. citizens, and non-US IMGs otherwise. These graduates form a significant part of the medical workforce globally but often encounter difficulties in securing training and employment in their chosen specialties, such as orthopaedic surgery [1].

The National Resident Matching Program (NRMP) plays a crucial role in the U.S. by matching medical school graduates with residency programs, particularly in competitive fields like orthopaedic surgery. In 2020, IMGs made up about 25% of all U.S. medical residents, more commonly in primary care than in surgical specialties [2]. In 2022, only 19 out of 875 orthopaedic surgery residency positions went to IMGs, constituting about 2.2% [3].

Several factors impact the low acceptance rate of IMGs into orthopaedic programs, including immigration policies, the applicants’ research outputs, and the perceived quality of their training [4]. This study aims to explore several aspects of IMGs in U.S. orthopaedic surgery residencies: comparing their match rates to those of U.S. DOs and MDs, identifying the geographic distribution of matched IMGs, pinpointing crucial factors for successful matching, and assessing the representation of non-US IMGs in these programs relative to the foreign-born U.S. population.

## Materials and methods

### Overview

This study was conducted using data from the NRMP from 2008 to 2022. The current study did not require approval from the ethics committee because it only used publicly available data and did not involve any human participants. IMGs were classified as either US or non-US citizens/permanent residents who attended an international medical school.

### Data source

The “Results and Data,” “Results by State, Specialty, and Applicant Type,” and documents available on the NRMP website and archives from 2008 to 2022 were reviewed [2, 3]. The available data for orthopaedic residency positions starting postgraduate year one (PGY-1) were included. Data from “Charting Outcomes in the Match” documents for the “International Medical Graduates” were obtained for years 2016, 2018, 2020, and 2022. NRMP data was evaluated for each state, except for Alaska, Delaware, Idaho, Maine, Montana, South Dakota, and Wyoming which did not have orthopaedic surgery residency training programs.

Because the number of IMGs who participated in the match program are not provided by NRMP, they were extracted from the 2018–2022 Electronic Residency Application Service (ERAS) statistics section maintained by American Association of Medical Colleges (AAMC) [5]. Data from the latest (2021) American Community Survey (ACS), one Year Estimates, were used to calculate the proportion of foreign-born people in each region [6].

### Variables

The number of participating programs, the number of filled and available positions, and their proportion (the overall “match rate”), as well as the percentage of positions filled by US and non-US IMGs were all evaluated. The three Canadian school graduate applicants were included in the US medical school graduates or seniors (US MDs) group.

Data on Step 1 and Step 2 clinical knowledge (CK) scores, number of abstracts, publications, and presentations were collected from “Charting Outcomes in the Match” reports for both US and Non-US IMGs. It should be noted that these reports are provided by the NRMP for the applicants who both consented to the use of their information and first-ranked the orthopaedic surgery program on their rank order list, excluding preliminary programs.

To assess geographical trends, the number of IMGs matched per state and year was extracted from the 2008–2022 NRMP reports. The average number of IMGs per program and year by state was calculated over a fifteen-year period.

To evaluate geographical variations in the US census divisions, state foreign-born and native population data was aggregated and grouped into nine US Census Bureau divisions. Puerto Rico was classified as part of the South Atlantic region.

### Statistical analysis

The Statistical Package for the Social Sciences (SPSS) version 26.0 was used for statistical analysis (IBM, Armonk, New York). The compound annual growth rate (CAGR) for the number of positions and programs during the study period was calculated using the CAGR formula with n as the year number: $\:{\left(\frac{Ending\:value}{Beginning\:value}\right)}^{(1/n)}-1.$

The matching rate was calculated by dividing the number of matched individuals in orthopaedic surgery residency by the number of open positions. Annual trends were calculated using a simple linear regression model, with a p value of < 0.05 considered significant.

To report the characteristics of matched and unmatched applicants, a weighted average was calculated for all available data in years 2016, 2018, 2020, and 2022.

The data from each state was aggregated and categorized into nine US census divisions as defined by the US Census Bureau [7]. For the purposes of this analysis, Puerto Rico was grouped with the South Atlantic region.

Tableau 2019.4.4 was used to geocode the states and census divisions. To assess for differences between the proportion of matched non-US IMGs (2008–2022) and US foreign-born population a simple ratio was calculated.

## Results

### Number of applicants and overall trends of match rates for US MDs, DOs, and IMGs

According to the ERAS 2018–2022 data, the total number of ERAS applicants for orthopaedic surgery was 8,168: 5,842 US MDs, 1,179 US DOs and 1,147 IMGs. Of these, 3,641 US MDs (62.32%), 372 US DOs (31.55%), and 62 IMGs (5.41%) matched through NRMP.

**Fig. 1:**
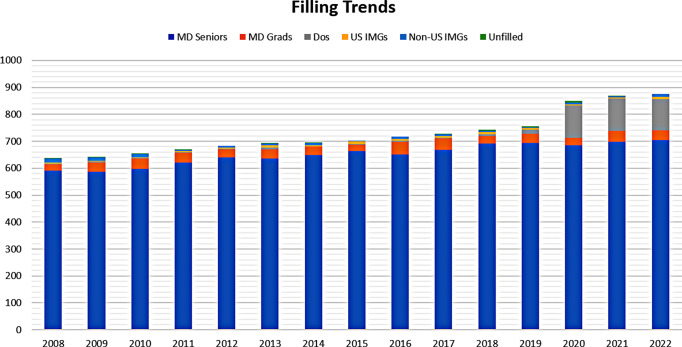
Trends in filling of the orthopaedic surgery residency positions from 2008 to 2022

The number of orthopaedic residency programs participating in the NRMP match increased from 160 (2008) to 210 (2022) (CAGR 1.71%). The total number of orthopaedic residency positions increased from 636 (2008) to 875 (2022) (CAGR 2.01%). The overall “match rate” for orthopaedic surgery positions was always equal to or greater than 99.5% during the study period (Fig. 1).

Among the filled spots, the proportion of positions filled by US MDs has declined from 96.69% in 2008 to 84.69% in 2022, a negative trend that was statistically significant at *P* = 0.005 with a beta coefficient of − 0.008. The proportion of positions filled by DO graduates and seniors increased from 0.31% in 2008 to 13.14% in 2022, a positive trend which was statistically significant at *P* = 0.003 with a beta coefficient of 0.009.

**Fig. 2:**
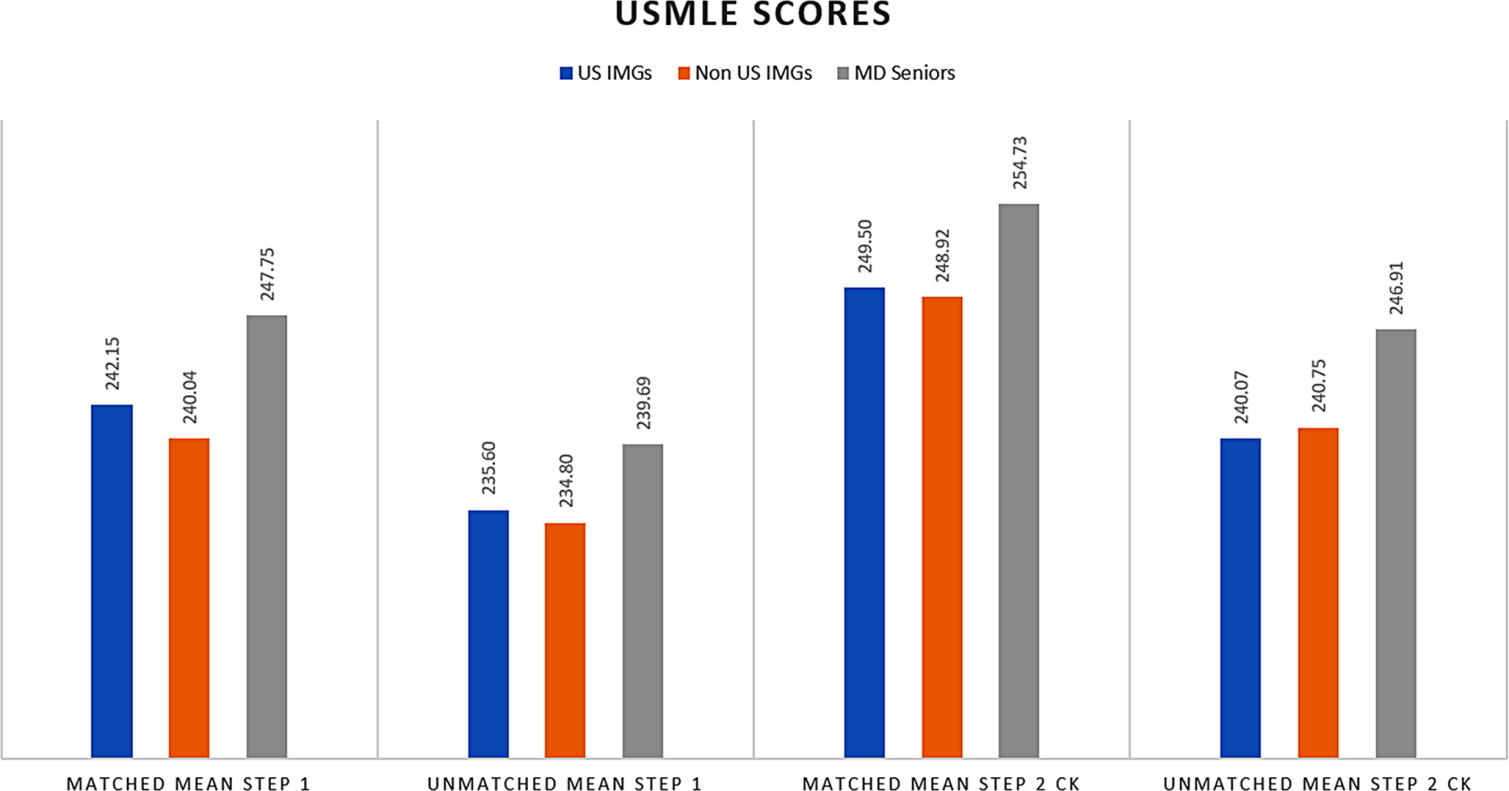
The mean Step scores of the studied groups for the years 2016, 2018, 2020, and 2022

The proportion of US IMGs has been constant over time, from 0.94% in 2008 to 0.91% in 2022, which did not show any significant change (*P* = 0.702). Alternatively, the proportion of non-US IMGs decreased significantly (*P* = 0.001, beta = -0.001) from 2.04% in 2008 to 1.26% in 2022 (Fig. 1). The overall IMG fill rate has also decreased from 2.04% in 2008 to 1.26% in 2022 (Fig. 2), a negative trend that was statistically significant at *P* = 0.002 with a beta coefficient of -0.001 (Fig. 1). Cumulatively over the study period (2008 to 2022), 91 US IMGs and 104 non-US IMGs have matched into orthopaedic surgery residency, a ratio of 0.875:1.

### Scores and publications

As shown in Fig. 2, for the years 2016, 2018, 2020, and 2022 the mean Step 1 scores of matched US IMGs and Non-US IMGs were 242.15 and 240.04, while the mean Step 2 CK scores were 249.50 and 248.92, respectively. The statistical difference could not be determined because raw data and standard deviations were unavailable.

Table 1 summarizes the average number of abstracts/publications for US IMGs, Non-US IMGs, and MD seniors for the years 2016, 2018, 2020, and 2022. The number of abstract/publications of matched US IMGs was at least three times higher than that of matched MD seniors and approximately two times higher than that of unmatched US IMGs. This proportion becomes more notable in matched non-US IMGs with abstract/publication numbers approximately five and three times greater than matched MD seniors and unmatched non-US IMGs, respectively.

**Table 1:** Characteristics of matched and unmatched applicants

	Matched			Unmatched		
	US IMG	Non-US IMG	MD Senior	US IMG	Non-US IMG	MD Senior
Abstracts/Publications	38.77	62.25	12.54	20.29	21.44	9.87
Research Experiences	7.00	7.72	5.19	6.88	3.49	4.99

**Fig. 3:**
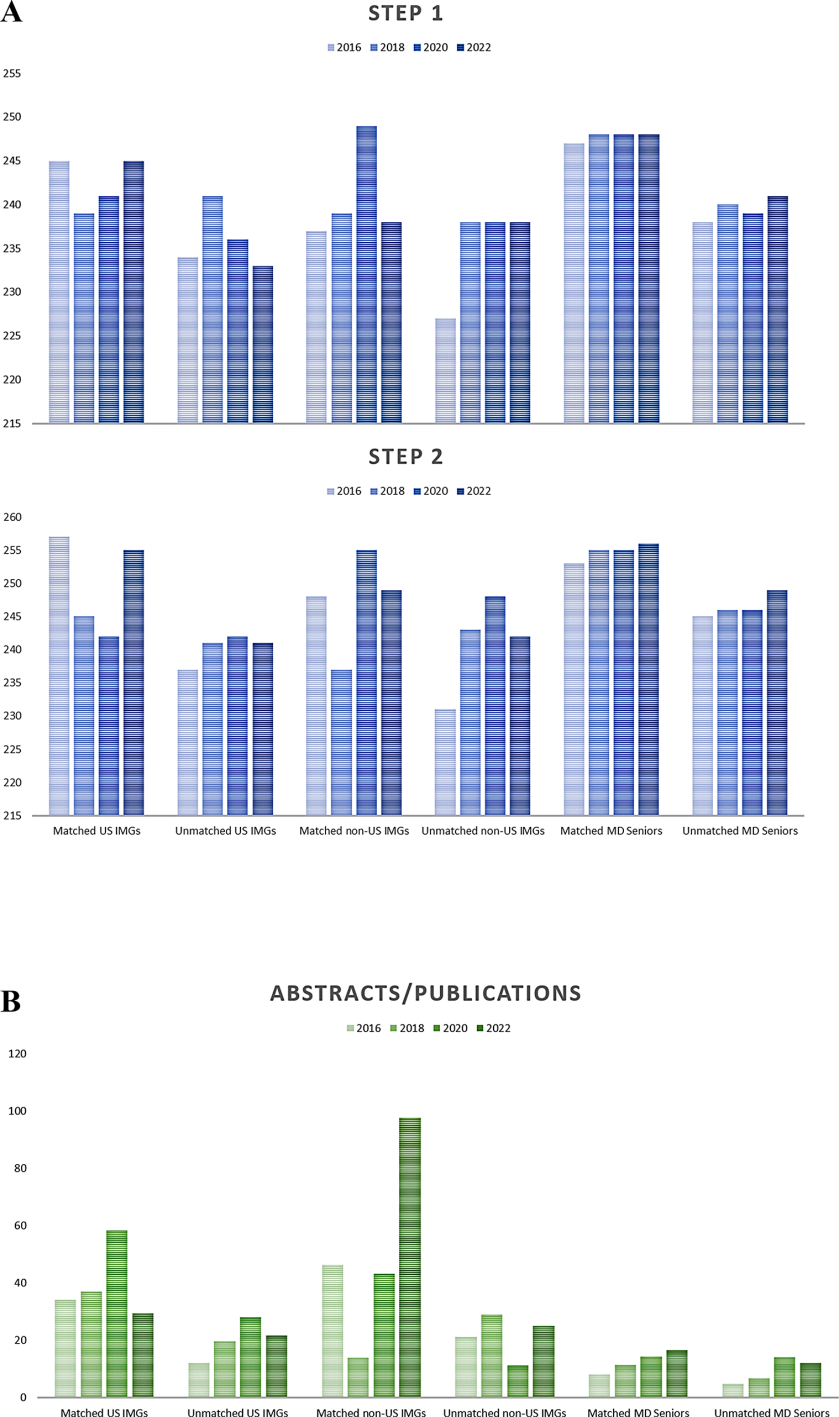
Trends in IMGs’ mean USMLE scores and abstract/publication numbers in 2016, 2018, 2020, and 2022. **A** USMLE step I and II, **B** Abstract/publication numbers

Figure 3 depicts the trends in IMGs’ mean USMLE scores and abstract/publication numbers in 2016, 2018, 2020, and 2022. During this time, the number of abstracts and publications among the matched groups generally increased. Matched non-US IMGs had the highest increasing trend, with a 110.3 to 597.1% increase in the number of abstracts and publications in 2022 compared to previous years with available data. In the years 2022, 2020, 2018, and 2016, the average number of abstracts and publications of non-US IMG was 97.6, 43.3, 14, and 46.4, respectively.

### Geographical trends across the states and variations in the US census division

**Fig. 4:**
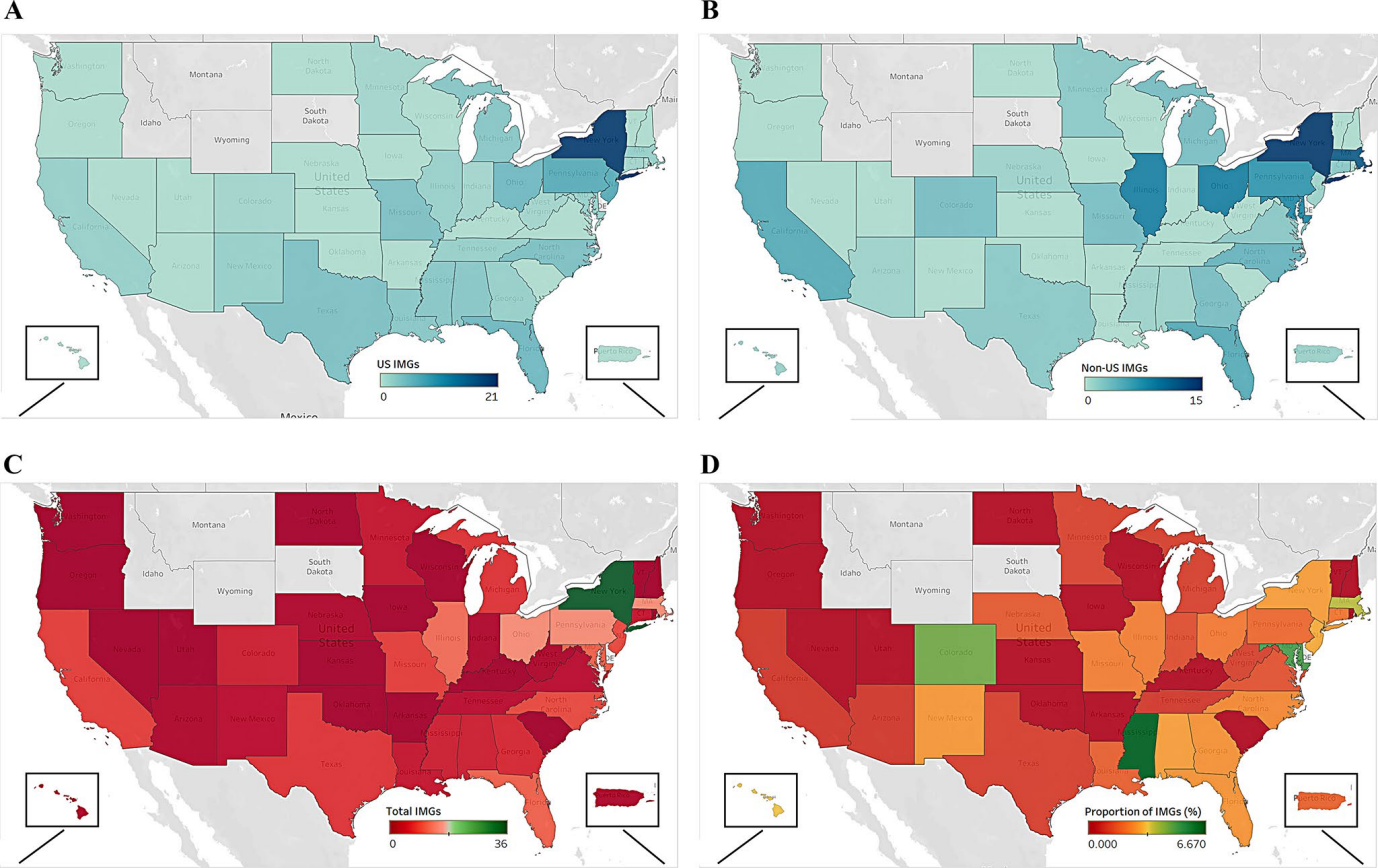
IMG matching status across the states from 2008 to 2022. (**A**) Number of matched US IMGs (**B**) Number of matched non-US IMGs (**C**) Total number of matched IMGs (**D**) The proportion of filled positions by IMGs (%)

Among the 43 states with orthopaedic surgery programs, the District of Columbia (D.C), and Puerto Rico, 16 (35.56%) of the regions had no IMGs during the previous 15 years. During the study period, New York, Pennsylvania, Ohio, and Massachusetts matched the most IMG physicians, matching more than thirteen IMG residents. Mississippi, Maryland, and Colorado were the most IMG-friendly states, with 6.7%, 5.1%, and 4.6% IMG to all filled positions ratios, respectively (Fig. 4 and Table 2).

**Table 2:** IMG matching status across the States from 2008 to 2022

State	Proportion of positions filled by IMGs (%)	US IMG number	Non-US IMG number	State	Proportion of positions filled by IMGs (%)	US IMG number	Non-US IMG number
**Alabama**	2.8	3	1	**Nevada**	0.0	0	0
**Arizona**	0.8	0	1	**New Hampshire**	0.0	0	0
**Arkansas**	0.0	0	0	**New Jersey**	3.0	7	1
**California**	0.8	2	5	**New Mexico**	2.7	2	0
**Colorado**	4.6	1	3	**New York**	2.8	21	15
**Connecticut**	2.1	1	2	**North Carolina**	2.4	4	4
**District of Columbia**	3.3	3	3	**North Dakota**	0.0	0	0
**Florida**	2.6	5	5	**Ohio**	2.1	5	9
**Georgia**	2.6	2	3	**Oklahoma**	0.0	0	0
**Hawaii**	3.3	0	1	**Oregon**	0.0	0	0
**Illinois**	2.2	2	9	**Pennsylvania**	1.8	7	7
**Indiana**	1.2	1	0	**Puerto Rico**	1.7	0	1
**Iowa**	0.0	0	0	**Rhode Island**	0.0	0	0
**Kansas**	0.0	0	0	**South Carolina**	0.0	0	0
**Kentucky**	0.0	0	0	**Tennessee**	0.8	2	0
**Louisiana**	1.6	3	0	**Texas**	0.9	4	2
**Maryland**	5.1	2	8	**Utah**	0.0	0	0
**Massachusetts**	3.7	2	12	**Vermont**	0.0	0	0
**Michigan**	1.0	3	3	**Virginia**	1.4	0	2
**Minnesota**	1.0	1	2	**Washington**	0.0	0	0
**Mississippi**	6.7	3	1	**West Virginia**	1.0	1	0
**Missouri**	2.2	4	3	**Wisconsin**	0.0	0	0
**Nebraska**	1.5	0	1				

IMG physicians were matched into orthopaedic surgery positions unevenly across the nine US census divisions. As shown in Table 3, the Middle Atlantic region had the highest percentage of IMGs matching into orthopaedic surgery residency (29.74%), while the Mountain region had the lowest (3.59%).

**Table 3:** IMG matching status across the census divisions from 2008 to 2022

	Geographic Region	Proportion of positions filled by IMGs (%)	US IMGs number	Non-US IMGs number	Division’s share of total IMGs (%)
Northeast Region	New England	2.37	3	14	8.72
	Middle Atlantic	2.46	35	23	29.74
Midwest Region	East North Central	1.59	11	21	16.41
	West North Central	1.21	5	6	5.64
South Region	South Atlantic	2.47	17	26	22.05
	East South Central	1.71	8	2	5.13
	West South Central	0.89	7	2	4.62
West Region	Mountain	1.75	3	4	3.59
	Pacific	0.70	2	6	4.10
	Total	1.79	91	104	100.00

### Comparison of foreign-born US population to non-US IMG physicians

According to 2021 ACS data, the combined population of D.C., Puerto Rico, and the 43 US states with orthopaedic surgery residencies was 327,569,652, with 44,950,622 (13.72%) being foreign born. Over the 15-year study period, non-US IMG physicians made up 0.96% of incoming PGY-1 orthopaedic surgery residents. Table 4 compares the foreign-born population within census divisions in the United States to the number of non-US IMG physician orthopaedic surgery residents within the census division. Non-US IMGs were under-represented in all nine geographic divisions when compared to the proportion of foreign-born people living within that division, particularly in the West South Central and Pacific regions.

**Table 4:** Comparison of proportion of matched non-US IMGs (2008–2022) and US foreign-born population in ACS 2021, by US census region

Geographic Region	A: Proportion of US Foreign born (%)	Total Population	B: Proportion of Non-US IMG (%)	Total Matched Residents	A: B
New England	**14.91**	13,720,492	**1.96**	716	7.62
Middle Atlantic	**17.83**	42,067,099	**0.98**	2354	18.25
East North Central	**7.95**	47,204,190	**1.04**	2012	7.62
West North Central	**6.23**	20,741,878	**0.66**	909	9.44
South Atlantic	**13.20**	68,846,557	**1.49**	1743	8.85
East South Central	**4.07**	19,474,454	**0.34**	586	11.92
West South Central	**13.76**	41,164,518	**0.20**	1012	69.64
Mountain	**11.70**	21,686,228	**1.00**	401	11.73
Pacific	**23.31**	52,664,236	**0.52**	1151	44.72
Total	**13.72**	327,569,652	**0.96**	10,884	**14.36**

## Discussion

Orthopaedic surgery is traditionally regarded as one of the most competitive specialties in the United States [3, 8], a fact underscored in our findings by more than half of applicants being unable to match during the previous five years. When compared to US MDs, IMG applicants have a much lower chance of acceptance (5.41% vs. 62.32% matching rate). Although the proportion of positions filled by US MDs has decreased significantly over the last 15 years, this was offset by an increase in US DO applicants, most likely as a result of the ACGME/AOA Single Accreditation System change in which all MD and DO programs are now ACGME-accredited [9], while the IMG proportion has remained consistent over time.

Given the low matching rate for IMGs, identifying factors contributing to a successful match is essential. Matched applicants generally had higher USMLE step scores than unmatched ones, yet matched IMGs scored lower than matched MD seniors, suggesting USMLE scores play a lesser role in IMG applications. The 2022 Program Directors’ Survey reported that about 65% of programs don’t require a target USMLE Step 2 score for IMGs, compared to 50% for US MD applicants [3]. With USMLE Step 1 now a pass/fail system [10], its historical data are less relevant. Although it is well known that USMLE scores are not a reliable predictor of resident performance [11–13], the recent decision to make USMLE Step 1 a pass-fail exam may increase the importance of Step 2 scores [14], especially for IMGs.

The publication number was also a noticeable criterion. With the shift to pass/fail for USMLE Step 1, the importance of research output has likely increased [15]. Recent trends show a “research fever,” with a notable rise in publications among matched MD seniors, especially in competitive specialties like orthopaedic surgery [16]. Interestingly, we found matched non-US and US IMGs had three to five times more publications than matched MD seniors. This high publication rate might reflect either a stronger inclination towards research among IMGs or heightened program expectations. Furthermore, the higher number of IMG publications could be attributed to the fact that many IMGs do multiple research years with clinical rotations to become matched into the US healthcare system before applying for a residency program [17]. However, medical educators and directors need to consider if the focus on research compromises other critical competencies, as success in the medical field demands skills beyond those needed for research [18]. Excessive research expectations can also lead to emotional exhaustion and burnout [19].

Geographical region also plays a role. Our research shows that New York, Pennsylvania, Ohio, and Massachusetts have consistently matched the most IMGs over the past 15 years. IMGs might benefit from applying to programs in these historically welcoming regions, especially considering the significant interview costs, estimated at $3,656 per applican [20]. The introduction of the Preference Signaling Program (PSP) for orthopaedic residency applications underscores the importance of such prioritization by enabling applicants to signal their preferences more effectively. Despite this, most program directors are skeptical that the PSP will enhance diversity [21]. Previous studies indicate that about 50% of orthopaedic residents match within the same U.S. census division as their medical school [22], a trend not applicable to IMGs trained abroad. Instead, the research fellowships abroad might replicate the role of U.S. medical schools. This is supported by findings that most research positions are in the northeast and south [23], coinciding with the areas hosting the most IMGs in our study. Future research should examine where IMGs conduct their U.S. research and its correlation with their match locations to provide clearer insights.

Conversely, it is equally important to explore the factors contributing to this low matching rate. The program directors’ reluctance to include IMGs in interviews may be one of the factors contributing to their lower match rate. According to the 2022 Program Directors’ Survey, approximately 98% of programs “never” or “seldom” interviewed IMGs. Another factor could be the role of visa requests, which plays a substantial role in applicant rankings [3].

Non-US IMGs who are not U.S. citizens need visa sponsorship unless they possess a green card. Typically, they have two visa options: (A) Exchange Visitor Visa (J-1 visa) sponsored by the Educational Commission for Foreign Medical Graduates (ECFMG) for up to seven years [24, 25], or (B) H1B visa for up to six years, sponsored directly by the hiring institutions, which requires lawyer fees and filing responsibilities [24, 26]. Consequently, the J-1 visa is a prevalent choice for non-green-card-holding non-US IMGs. ERAS 2021 reports indicate that ECFMG sponsored over 13,000 J-1 visas, with orthopaedic surgery residencies making up less than 1% of these [25].

Medical residents on J-1 visas must return to their home country for two years before they can work in the U.S. or undertake a waiver job in medically underserved areas for three years [24, 27]. This requirement offers an opportunity for the U.S. healthcare system to expand its service to underserved communities through the skills of IMGs, who bring understanding of health disparities and cross-cultural expertise [28]. However, our findings highlight a significant underrepresentation of non-US IMGs, particularly in the West South Central and Pacific divisions. Additionally, our research showed that over one-third of U.S. states have not matched any IMGs in the last 15 years. A previous study found that over half of physicians began practicing within 75 miles of their residency site [29], potentially suggesting that the lack of IMG matching might lead to a shortage of diverse perspectives within the U.S. healthcare workforce.

Diversity in medicine enhances patient care and education, yet orthopaedic surgery lags behind [30–32]. In July 2019, the ACGME revised its Common Program Requirements to promote diverse, inclusive resident recruitment and retention [33]. Diversity encompasses factors like gender, race, culture, origin, and training place. However, the impact on IMGs remains unclear [34]. Historically, orthopaedic surgery had few female residents and slow gender diversity growth [35]. Recent research by Poon et al. [36] indicated race, not gender, affects orthopaedic residency matches, but did not mention IMGs, whose role in diversity is under-recognized.

This study provides a comprehensive analysis of orthopaedic surgery residency matching trends for IMGs, with certain limitations such as incomplete NRMP data and reliance on AAMC for participant numbers. Constraints on applicant responses to the NRMP may also affect the representation of our results. The study did not explore the impact of the origins of IMGs’ degrees over 15 years, nor did it delve into network connections or program-level ‘friendliness’ towards IMGs, which varies by state and could overshadow attitudes of individual programs.

## Conclusion

Matching into orthopaedic surgery residencies is highly competitive for IMGs. A significant mismatch exists between the foreign-born population and non-US IMG orthopaedic residents, particularly in the West South Central and Pacific regions. Key trends suggest that higher publication numbers enhance matching chances, with the Northeast being a more favourable region for IMGs.
